# Inflammasome modulation with P2X7 inhibitor A438079-loaded dressings for diabetic wound healing

**DOI:** 10.3389/fimmu.2024.1340405

**Published:** 2024-02-15

**Authors:** Jordan R. Yaron, Selin Bakkaloglu, Nicole A. Grigaitis, Farhan H. Babur, Sophia Macko, Samantha Rhodes, Solenne Norvor-Davis, Kaushal Rege

**Affiliations:** ^1^ Center for Biomaterials Innovation and Translation, The Biodesign Institute, Arizona State University, Tempe, AZ, United States; ^2^ School for Engineering of Matter, Transport & Energy, Arizona State University, Tempe, AZ, United States; ^3^ Biological Design Graduate Program, Arizona State University, Tempe, AZ, United States; ^4^ Chemical Engineering, Arizona State University, Tempe, AZ, United States

**Keywords:** wound dressing, silk fibroin, inflammasome, small molecule, drug delivery, wound healing

## Abstract

The inflammasome is a multiprotein complex critical for the innate immune response to injury. Inflammasome activation initiates healthy wound healing, but comorbidities with poor healing, including diabetes, exhibit pathologic, sustained activation with delayed resolution that prevents healing progression. In prior work, we reported the allosteric P2X7 antagonist A438079 inhibits extracellular ATP-evoked NLRP3 signaling by preventing ion flux, mitochondrial reactive oxygen species generation, NLRP3 assembly, mature IL-1β release, and pyroptosis. However, the short half-life *in vivo* limits clinical translation of this promising molecule. Here, we develop a controlled release scaffold to deliver A438079 as an inflammasome-modulating wound dressing for applications in poorly healing wounds. We fabricated and characterized tunable thickness, long-lasting silk fibroin dressings and evaluated A438079 loading and release kinetics. We characterized A438079-loaded silk dressings *in vitro* by measuring IL-1β release and inflammasome assembly by perinuclear ASC speck formation. We further evaluated the performance of A438079-loaded silk dressings in a full-thickness model of wound healing in genetically diabetic mice and observed acceleration of wound closure by 10 days post-wounding with reduced levels of IL-1β at the wound edge. This work provides a proof-of-principle for translating pharmacologic inhibition of ATP-induced inflammation in diabetic wounds and represents a novel approach to therapeutically targeting a dysregulated mechanism in diabetic wound impairment.

## 1 Introduction

Chronic cutaneous wounds are a major medical burden. More than 6 million chronic wound cases amount to a cost of over $20 billion per year in healthcare costs in the USA - nearly 5% of the total cost of Medicare and Medicaid (1). Acute wound healing proceeds along a spectrum of continuous and overlapping phases of (i) hemostasis, (ii) inflammation, (iii) proliferation and (iv) remodeling (2–4). Delay in the onset or resolution of any stage leads to impaired healing and complex comorbidities such as diabetes are commonly associated with poor healing. Diabetic patients have a 15% lifetime risk for chronic foot ulcers, which remain the primary cause for amputation and result in significant negative emotional, physical, and financial costs (5). The 5-year survival for lower limb amputations due to diabetic wounds is only 23%, approximately the same as the mortality rate seen for all cancers (6). Selective targeting of dysregulated repair mechanisms in diabetic wounds may provide an effective approach to reverting impaired healing back to a healthy, acute healing state and is an active field of investigation (7).

While inflammation is known to be important for initiating reparative events in healing wounds (8), diabetic wounds are characterized by dysregulated and sustained inflammation (9). The inflammasome is a multi-protein platform that drives inflammatory responses in a wide and growing array of infected and sterile pathologies (10). The NLRP3 inflammasome is the most widely studied of the various inflammasome platforms in part due to its role in the greatest number of identified pathologies and is most dominantly active in myeloid-lineage immune cells (11), though other cells have been shown to exhibit NLRP3 pathway activity (12–15). The NLRP3 inflammasome can be regarded as a central nexus to cellular stressors upon myeloid cells and, once licensed for activation by a priming signal such as interleukin (IL)-1α or lipopolysaccharide (LPS), is activated by diverse stimuli such as ion flux induced by extracellular damage associated molecular patterns (DAMPs), frustrated phagocytosis of crystalline structures, lysosomal instability, endocytic pathway disruption, and others (10, 16). While the NLRP3 inflammasome pathway has been shown to be an important early player in healthy wound healing, its sustained activation has been implicated in impaired wound healing in diabetes (17).

Detection of extracellular ATP by the purinergic receptor P2X7 is a canonical initiator of NLRP3 inflammasome activity (18). Targeting extracellular ATP-induced inflammasome activation has been reported to improve diabetic wound impairment. Mirza and colleagues were the first to report a role for the NLRP3 inflammasome in diabetic wound impairment using genetically diabetic mice (19). Using glyburide, an inhibitor of K_ATP_ potassium channel activity and extracellular ATP-induced NLRP3 activation, they showed an improvement in wound closure and a reduction in ongoing inflammation at 10 days post-wounding. Subsequently, Bitto and colleagues showed improved wound closure in diabetic mice with the P2X7 receptor antagonist Brilliant Blue G and the direct NLRP3 inhibitor Bay 11-7082 (20). In both studies, the inhibitors required multiple administrations owing to the short half-life of these molecules. Repeated administration of drugs to wounds can cause secondary injury, thus prolonging healing and increasing the risk of additional complications (21, 22). Further, multiple administrations of drugs or dressings reduce patient compliance to complex wound care regimens (23). Thus, dressings which are capable of sustained delivery of drugs may enable improved clinical care for wound care patients (24).

We have previously characterized the *in vitro* activity of A438079, a potent small molecule inhibitor of P2X7 (25), and reported that its use for P2X7 inhibition results in suppression of both potassium and calcium fluxes and abrogation of mROS generation thereby potently suppressing NLRP3 inflammasome activation in macrophages in culture (26). Here, we develop a biomaterial-based platform to act as a depot for sustained delivery of A438079 *in vivo* and evaluate its efficacy in a model of impaired diabetic wound healing. Prior reports indicate A438079 has a low bioavailability (19%), high plasma protein binding (84%), and short half-life (1.02 hours) (27). Because inflammasome activity persists for at least 3-6 days in diabetic wounds, repeated administration or sustained release of inflammasome-modulating drugs is necessitated for therapeutic augmentation. We describe the development and characterization of a wound dressing fabricated from purified silk fibroin, a naturally occurring biopolymer with high biocompatibility and amenability to physicochemical modification, that can be loaded with variable amounts of A438079 and exhibit sustained delivery kinetics compatible with modulation of early-stage over-activation of inflammasome activity in diabetic wounds. We first test our all-in-one drug-loaded dressings *in vitro* using mouse macrophages and then evaluate efficacy in a wound healing model *in vivo* in diabetic mice. To our knowledge, this is the first demonstration of a therapeutic topical dressing platform to target P2X7-mediated inflammation in diabetic wounds and presents a novel approach to therapeutically modulate impaired wound healing in different pathologies.

## 2 Materials and methods

### 2.1 Generation and characterization of silk fibroin films

Silk fibroin was obtained by degumming *Bombyx mori* silkworm cocoons as previously described (28). Briefly, raw cocoons were cut into small sections and washed thoroughly in nanopure water (resistivity 18.2 MΩ-cm). Degumming was performed by boiling washed cocoon pieces in a solution of Na_2_CO_3_ in nanopure water (0.005 g/mL, 0.5% w/v) for 30 minutes with slow stirring. Degummed material was washed three times in nanopure water for 15-20 minutes in each wash, pulled into thin sections, and dried overnight at room temperature. The dried silk fibroin material was dissolved in 9.4M lithium bromide at 60°C (2 g silk fibroin per 10 mL solution) with stirring by adding the silk piece wise. Once dissolved, the solution was continuously stirred for an additional 4 hours at 60°C. Dissolved silk solution was clarified through cotton filter paper, transferred to 3.5 kDa dialysis membranes, and dialyzed against nanopure water for 72 hours at 4°C. Concentration and weight percentage of resultant silk fibroin solution was determined by back-calculation of the mass of dried, known volume of silk in a pre-weighed weigh boat. Purified silk composition was characterized by FT-IR on a Thermo Nicolet 6700 spectrometer.

Films were cast in a 100 mm petri dish by dispensing 4 or 8 mL of an 8% w/v solution followed by overnight solvent evaporation at room temperature. Rheometry of 10x15-mm films was performed by Dynamic Mechanical Analysis on a Discovery HR 30 rheometer (TA Instruments) with a frequency sweep from 1 Hz to 10 Hz and a strain of 0.1% to measure storage and loss moduli. Round films were produced by using a 6-mm hollow punch tool. Films were made insoluble by placing in an autoclavable pouch and autoclaving in a 20-minute steam/heat cycle. Films were measured using a micrometer-type caliper (Rexbeti) to confirm thickness prior to use.

### 2.2 Scanning electron microscopy

Silk films were sputter coated with 1.5-2nm gold coating on a Cressington 108 sputter coater (exposure 120s with an argon gas environment). High-resolution field emission SEM was performed on an Auriga (Zeiss) scanning electron microscope equipped with a high-resolution Gemini Field Emission-SEM column and Schottky thermal field emitter operating at 5.00 kV HV and a monopole magnetic immersion final lens. Images were collected *en face* and in cross-section after cutting the film with a scalpel.

### 2.3 Film swelling and passive degradation studies

Autoclaved films were placed in 1X PBS and incubated for up to 40 days at room temperature. Film thickness was measured with a micrometer-type caliper (Rexbeti) and degradation was determined by protein quantity in solution at 40 days using a BCA protein assay (Pierce).

### 2.4 A438079 molecular docking

A438079 was docked to the published structure for P2X7 in the closed, apo state (PDB 5U1L) (29) using Webina, a webserver for Autodock Vina (30). Docking results were visualized in Chimera X version 1.3 (31). Results were validated against prior docking reported by Allsopp et al. (32).

### 2.5 A438079 spectroscopy analysis

A438079 was purchased from Santa Cruz Biotechnology and dissolved in a solution of 50% DMSO in nanopure water to produce a 25 mM stock solution. Aliquots were prepared and stored at -20°C. Two-fold serial dilutions from 2.5 mM to 0.04 mM were generated in 1X PBS and dispensed in 50 µL volumes in a UV-transparent 96-well plate. Absorbance spectroscopy was performed on a Biotek Synergy H2 plate reader from 200-300 nm with a 2 nm resolution. Diagnostic peaks were observed at 226 nm and 258 nm.

### 2.6 Loading and release studies

Insolubilized silk films were incubated in solutions of 1.25 mM A438079 for 24 hours. The amount of the small molecule inhibitor drug loaded was evaluated by absorbance spectroscopy of the post-loaded solution versus solution that was not incubated in the presence of a film. Release studies were performed by incubating a loaded film in 1X PBS at room temperature with shaking. At regular intervals, the PBS soaking solution was removed and analyzed by absorbance spectroscopy. The films were placed in fresh solutions of PBS to generate continuous sink conditions. Cumulative release was calculated and fit to a two phase burst release model in GraphPad Prism with the “two phase association” equation (33):


(1) $$\begin{array}{l} \text{SpanFast}=\left(\text{Plateau}-\text{Y}0\right)*\text{PercentFast}*.01\\ \text{SpanSlow}=\left(\text{Plateau}-\text{Y}0\right)*\left(100-\text{PercentFast}\right)*.01\\ \text{Y}=\text{Y}0+\text{SpanFast}*\left(1-\text{exp}\left(-\text{KFast}*\text{X}\right)\right)+\text{SpanSlow}*\left(1-\text{exp}\left(-\text{KSlow}*\text{X}\right)\right) \end{array}$$

### 2.7 Cell culture

J774DUAL cells (Invivogen) were cultured in DMEM with 10% FBS containing 1% penicillin/streptomycin and additional supplementation of selective antibiotics according to manufacturer’s procedure (5 µg/mL Blasticidin, 100 µg/mL Zeocin^®^). Cells were fed twice a week and passaged by cell scraping. Cell viability during passaging was evaluated by Trypan Blue staining using an EVE™ Plus Automated Cell Counter (NanoEnTek) to ensure 95% or greater viability prior to use.

### 2.8 IL-1β ELISA analyses

On day 1, J774DUAL cells were seeded at 10^5^ cells/well in a tissue culture-treated 96-well plate and incubated overnight at 37°C/5% CO_2_ in a humidified incubator in complete DMEM without selective antibiotics. On the same day, an ELISA plate (R&D systems, DY008) was coated with capture antibody to IL-1β (R&D Systems, DY401) according to manufacturer’s instructions, sealed with plate film, and incubated at room temperature overnight. On day 2, cells were either left untreated or primed with 1 µg/mL E. coli LPS (O111:B4; “first signal”) in 100 µL final volume fresh DMEM (no selective antibiotics) and returned to the incubator for 4 hours. Concurrently, A438079-loaded films were incubated in 220 µL fresh DMEM (10% FBS, 1% pen/strep, no selective antibiotics) at room temperature in a 1.5 mL microcentrifuge tube. ELISA plates were washed and blocked during the priming period according to manufacturer’s protocol. During the final 30 minutes of priming (3.5 hours after initial treatment with LPS), 50% (i.e., 50 µL) of the incubation medium was replaced with either fresh DMEM or film-conditioned medium. The plate was returned to the incubator for the final 30 minutes of priming. To stimulate inflammasome activation (“second signal”), cells were treated with 3 mM final concentration of extracellular ATP (3 µL of a 100 mM stock solution prepared in fresh DMEM mixed into 100 µL final culture volume) and returned to the incubator for 45 minutes. After 45 minutes, complete (100 µL) cell supernatants were collected, transferred to the blocked ELISA plate along with recombinant IL-1β standards, sealed with plate film, and incubated overnight at 4°C. On day 3, the ELISA plate was washed, probed with detection antibodies, and developed according to manufacturer’s procedure. The developed ELISA plate was read on a Biotek Synergy H2 plate reader at 450 nm with background subtraction at 540 nm. Data analysis using 4-parameter logarithmic regression was performed in GraphPad Prism.

### 2.9 Immunofluorescence microscopy

Cells were treated as in Section 2.8 above for ELISA analyses. After 45 minutes of ATP treatment, the culture medium was aspirated, and cells were fixed in 2% formaldehyde (freshly prepared from paraformaldehyde in 1X PBS) for 20 minutes at room temperature. Cells were permeabilized with 1X TBS containing 0.2% Tween-20 (TBST) for 20 minutes at room temperature and blocked in 5% BSA prepared in 1X TBST for 1 hour at room temperature. Cells were incubated overnight at 4°C with rabbit polyclonal antibody to ASC (AL177, 1:200 dilution, Adipogen) in 5% BSA/TBST. The following day, cells were washed with TBST and incubated with CF568-conjugated donkey anti-rabbit secondary antibody (1:500 dilution, Biotium) and iFluor 488-conjugated Phalloidin (1:1000 dilution, AAT Bioquest) for 2 hours at room temperature protected from light. Cells were washed with TBST and incubated for 30 minutes with 10 µg/mL DAPI (Abcam) in 1X PBS at room temperature protected from light. Cells were washed with PBS and submerged in 250 µL Fluoromount G (Thermo-Fisher) and stored at 4°C protected from light until imaging. Cells were imaged on a Nikon AXR confocal (22.1 µm pinhole) mounted to a Ti2 base with a Plan Apo 60× Oil NA 1.42 objective lens using 408, 488, and 561 nm laser lines paired to DAPI (429-474 nm), AF488 (503-541 nm), and AF568 (571-625 nm) emission windows, respectively, with at 1024x1024 resolution setting with 2X frame averaging. Images were collected with NIS-Elements AR software (ver. 5.41.01 build 1709) and analyzed using FIJI/ImageJ (ver. 2.14.0/1.54f).

### 2.10 *In vivo* wound healing studies

All animal procedures were approved by the Institutional Animal Care and Use Committee of Arizona State University under protocol #21-1830R. Mice were purchased from the Jackson Laboratory and kept on a standard 12/12 light-dark cycle in specific pathogen-free housing conditions and given food and water *ad libitum*. Full-thickness wound healing was performed in male and female 12-week old obese, diabetic “db/db” mice (BKS.Cg-*Dock7^m^* +/+ *Lepr^db^*/J; JAX strain code 000642) as previously described (34, 35). Briefly, non-fasting blood glucose was measured within one week of surgery by saphenous vein collection and confirmed to be >400 mg/dL using a glucometer. Mice were anesthetized and a 1x1-inch midline intrascapular area between the base of the neck and apex of the spine was shaved and sterilized with successive washes with alcohol and chlorhexidine gluconate solution. A 6-mm full-thickness biopsy punch was performed and wounds were either treated directly with 20 µL saline, 100 µM A438079 in 20 µL saline, empty silk film, or silk film loaded with A438079. Treatments were applied a single time during the study. A silicone splint (14mm OD x 7mm ID x 0.5mm thick, Grace Biolabs) covered with Tegaderm occlusive dressings (3M) were affixed using cyanoacrylate glue (Krazy Glue) and six interrupted sutures (4-0 black Ethilon monofilament with a FS-2 reverse cutting needle; Ethicon, Inc.). Mice were returned to single housed cages to prevent removal of splints by cage mates and monitored daily until euthanasia at 10 days post-wounding. Wound area (planimetry) after splint removal was documented by digital photography using a mobile phone and analyzed with calibration in ImageJ/FIJI.

### 2.11 Immunohistochemistry

Wound tissues were collected at day 10 post-wounding and fixed in 10% neutral-buffered formalin. Tissues were dehydrated through graded alcohol into xylene followed by paraffin perfusion. Tissues were embedded into paraffin blocks and 5-6 µm sections were captured onto positively charged glass slides. Sections were rehydrated and epitope retrieval was performed by incubation in sodium citrate buffer (10 mM, pH 6.0 with 0.05% Tween-20) at 60°C overnight. Sections were blocked with 5% bovine serum albumin (BSA) in TBS/0.1% Tween-20 for 1 hour at room temperature and incubated overnight with goat anti-mouse IL-1β primary antibody (R&D Systems, AF-401-NA, 1:100) in 5% BSA in TBS/0.1% Tween-20 at 4°C overnight. Sections were washed with TBS/0.1% Tween-20 and peroxide quenching was performed with 3% hydrogen peroxide in PBS for 15 minutes at room temperature followed by thorough washing. Sections were incubated in horseradish peroxidase (HRP)-conjugated donkey anti-goat secondary antibody (Jackson Immunoresearch #705-035-147, 1:500) for 2 hours at room temperature followed by thorough washing. Sections were developed with ImmPACT DAB substrate (Vector Labs #SK-4105) for 5 minutes, counterstained with hematoxylin (Gill No. 2, #GHS232, Sigma Aldrich), dehydrated, and mounted with CytoSeal XYL (Thermo Fisher). Slides were scanned at 40X magnification on an Olympus VS200 Slide Scanner and quantified using QuPath software v0.4.3 (36).

## 3 Results

### 3.1 Fabrication and characterization of insoluble, tunable thickness silk fibroin film dressings

**Figure 1:**
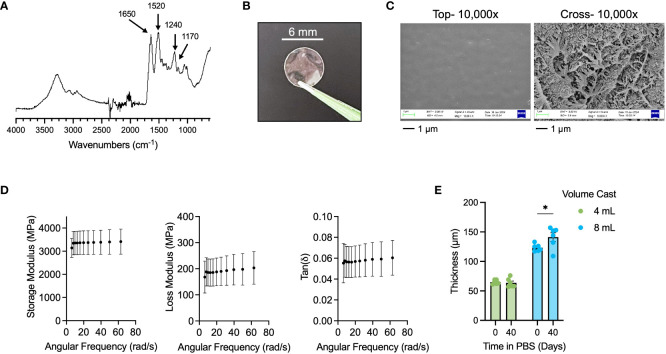
Fabrication and characterization of insoluble, tunable silk fibroin wound dressings. **(A)** FT-IR spectra of purified silk fibroin with key peaks indicated for amide I (1650 cm^-1^), amide II (1520 cm^-1^), and amide III (1240 cm^-1^). **(B)** Representative 6-mm diameter silk wound dressing. **(C)** SEM of top surface (“top”) and cross-section (“cross”) of insoluble silk dressing at 10,000x. Scale bar = 1 µm. **(D)** DMA results for Storage Modulus, Loss Modulus, and Tan(δ) of insoluble silk dressing. **(E)** 40-day swelling study of 4 mL (60-µm thick) and 8 mL (120-µm thick) cast silk dressings. Results are shown as mean ± standard error. Statistics are performed by two-way ANOVA with Fisher’s LSD. *p<0.05.

We sought to generate a tunable thickness, insoluble silk fibroin film as a long-lasting wound dressing matrix. We enriched silk fibroin from *Bombyx mori* silkworm cocoons and characterized the polypeptide by FT-IR spectroscopy (**Figure 1A**) with expected amide I peak at 1650, amide II peak at 1520, and amide III peak at 1240 cm^-1^ (37). We cast 100-mm diameter sheets of silk fibroin in petri dishes and generated films by solvent evaporation at room temperature overnight. Using a hollow punch tool, we fabricated 6-mm diameter wound dressings (**Figure 1B**), which we made insoluble by autoclaving (38). Autoclaving is thought to increase the beta sheet content in silk fibroin, which makes them resistant to dissolution in aqueous solutions (39). We performed scanning electron microscopy (SEM; **Figure 1C**) on insolubilized films and observed that while the exposed surfaces of the films were relatively smooth and without notable features, the cross-section of the films indicated a highly complex network structure. Dynamic mechanical analysis of the films (**Figure 1D**) indicated that the films exhibited durable elastic properties with high storage and loss moduli of ~3300 MPa and ~200 MPa, respectively, with a low tan(δ) of ~0.06, which indicated elastic nature of these films. Film thickness was tuned by modulating the amount of silk solution used to cast the initial film sheet, with 4 mL of 8 w/v% silk solution in a 100-mm petri dish resulting in films of ~60-µm in thickness, and 8 mL of 8 w/v% silk solution resulting in films of ~120-µm thickness (**Figure 1E**). We evaluated the passive degradation of the films in PBS over a period for 40 days at room temperature and found that 60-µm films cast from 4 mL solutions did not exhibit any swelling behavior (-2% swelling ratio), while 120-µm films cast from 8 mL solutions exhibited minor swelling behavior to ~140-µm (16.7% swelling ratio). In both cases, BCA protein assay of the incubation solution indicated no presence of dissolved protein (data not shown) and thus no passive degradation over 40 days. Taken together, we generated insoluble and durable, tunable thickness silk fibroin films appropriate for wound dressing applications (40, 41).

### 3.2 Characterization and drug release characterization of P2X7 receptor inhibitor A438079

**Figure 2:**
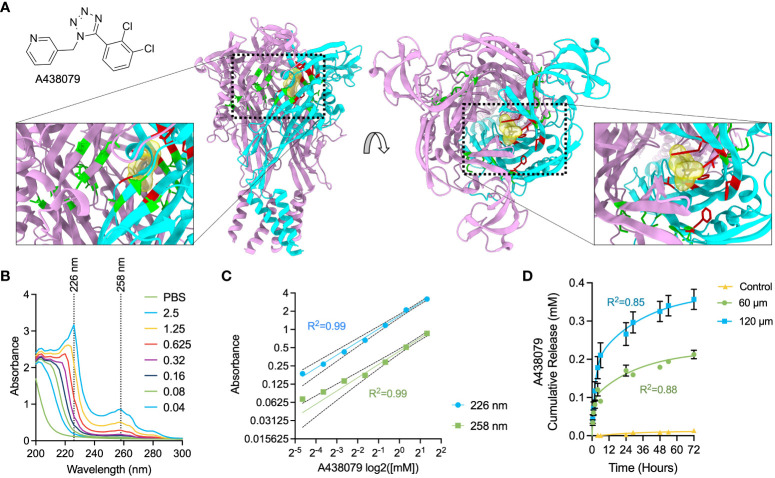
Characterization of A438079 cargo and drug delivery kinetics. **(A)** Chemical structure of A438079 and illustration of molecular docking region in the P2X7 structure in top and side view. ATP-binding pocket residues are colored green, while A438079-binding residues are colored red. One monomeric unit of P2X7 is colored cyan. **(B)** Spectrophotometric analysis of A438079 with diagnostic wavelengths indicated at 226 nm and 258 nm. Values in the legend are given as [A438079] in mM. **(C)** Log-log concentration versus absorbance for A438079 at diagnostic wavelengths of 226 nm and 258 nm demonstrating linear response with 95% confidence intervals and R^2^ of regression indicated. **(D)** Release kinetics of A438079 over 72 hours at room temperature in 1X PBS from 60-µm thick and 120-µm thick films versus unloaded 60-µm thick films (control) with curve fit for two phase burst release model results. R^2^ given for model fit. Results are shown as mean ± standard error.

A438079 is a water-insoluble, selective antagonist of the P2X7 purinergic receptor that allosterically binds in a region of the ion-permeating channel in the left flipper of the extracellular domain in close proximity to the previously described residues F88, F92, T94, F95, and F103 (**Figure 2A**) (32, 42). This allosteric site is ideal to facilitate druggability of the P2X7 receptor, as allosteric sites are commonly more selective, allow lower target-based toxicity, fewer side effects, and accessible physicochemical properties (43, 44). We previously investigated the inhibitory function of A438079 and reported a potent ability to ameliorate extracellular ATP-evoked potassium and calcium flux and subsequent inflammasome activation in mouse macrophages (26). We performed UV-VIS spectrophotometric analysis of A438079 in PBS (**Figure 2B**) and identified two diagnostic absorption peaks at 226 nm and 258 nm which exhibit linear response from 40 µM to 2500 µM (**Figure 2C**). We passively loaded 60-µm and 120-µm films with A438079 overnight in a 1.25 mM loading solution in PBS and measured release over 72 hours into PBS. Kinetics analysis indicated a release profile fitting to a two-phase burst release model (Equation 1), with release amount proportional to film thickness (**Figure 2D**). A concentration of approximately 100 µM and 200 µM in 100 µL PBS was measured in the first 6 hours from 60-µm and 120-µm films, respectively. Release continued at ~40 µM and ~60 µM per day for the first 2 days for 60-µm and 120-µm films, slowing to ~20 µM and ~30 µM by the third day, respectively. We observed that prolonging the drug loading time from 1 days to 3 days increased the amount of released drug, indicating a one-day load does not saturate loading capacity of 60-µm films (**Supplementary Figure S1**). Films were stored dry in plastic bags after loading and kept at room temperature for up to 1 week prior to use. Thus, insoluble silk fibroin wound dressings were loaded with A438079 in a film-tunable and loading-tunable manner with differential release kinetics into aqueous solution.

### 3.3 *In vitro* evaluation of A438079-loaded films

**Figure 3:**
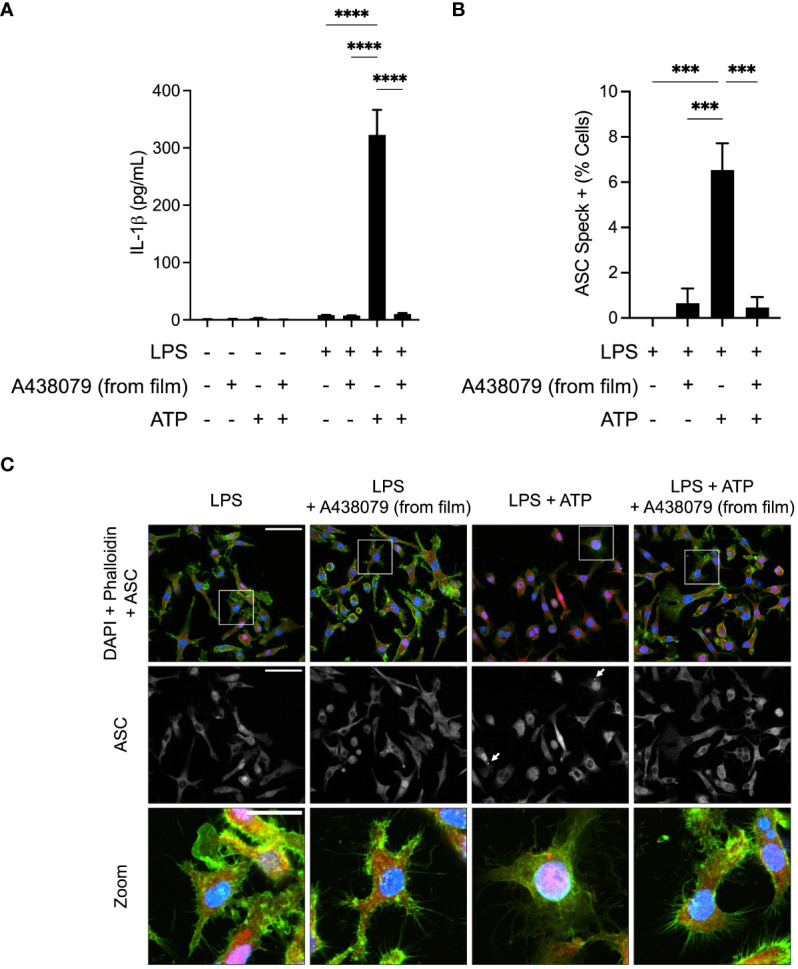
*In vitro* evaluation of A438079-loaded films. **(A)** ELISA quantification of supernatant IL-1β released from J774DUAL macrophages left untreated or primed for 4 hours with 1 µg/mL LPS and stimulated for 45 minutes with 3 mM ATP. Cells were treated for the final 30 minutes of priming with conditioned media exposed to A438079-loaded silk films for 4 hours. Results are given as mean ± standard error with statistics calculated by two-way ANOVA and Fisher’s LSD. ****p<0.0001. **(B)** Quantification of perinuclear ASC specks from LPS-primed J774DUAL macrophages stimulated for 45 minutes with 3 mM ATP with or without 30 minutes pre-treatment with conditioned media exposed to A438079-loaded silk films. Results are given as mean ± standard error with statistics calculated by one-way ANOVA with Fisher’s LSD. ***p<0.001. **(C)** Representative confocal micrographs of J774DUAL macrophages immunostained for ASC (red) and stained for DNA with DAPI (blue) and F-actin with phalloidin (green). Perinuclear ASC specks are indicated with white arrows and shown with enhanced visibility in zoomed fields. Scale bars represent 50 µm in the first two rows and 25 µm in the zoomed field.

We next sought to determine whether A438079-loaded films would exhibit therapeutic properties in an *in vitro* system of inflammation. We used the mouse macrophage cell line, J774DUAL (derivative of J774A.1), to investigate inflammasome signaling. We first performed ELISA to measure production and release of IL-1β, a key inflammatory cytokine and secreted inflammasome mediator (**Figure 3A**). J774DUAL cells primed with LPS for 4 hours with E. coli LPS and stimulated for 45 minutes with 3 mM extracellular ATP to activate P2X7 receptor signaling robustly secreted IL-1β. When cells were treated with conditioned medium exposed to A438079-loaded silk films, near-complete inhibition of IL-1β secretion was observed. We next evaluated whether this inhibition of IL-1β secretion was due to inhibition of inflammasome activity. We performed immunofluorescence of J774DUAL cells primed with LPS and stimulated with 3 mM extracellular ATP to evaluate the formation of perinuclear ASC specks, a canonical indicator of inflammasome assembly (**Figures 3B, C**). Without inhibitor treatment, approximately 6% of the population exhibited perinuclear ASC specks. We note these levels of ASC speck positivity are in agreement with prior work by us (26) and others (45, 46), but are likely an underestimation due to repeated wash steps during immunofluorescence processing. When treated with conditioned medium exposed to A438079-loaded silk films, <0.5% of the population exhibited ASC specks. Thus, A438079-loaded silk films robustly inhibit inflammasome assembly and activity when evaluated with mouse macrophages *in vitro*.

### 3.4 *In vivo* evaluation of A438079-loaded films

**Figure 4:**
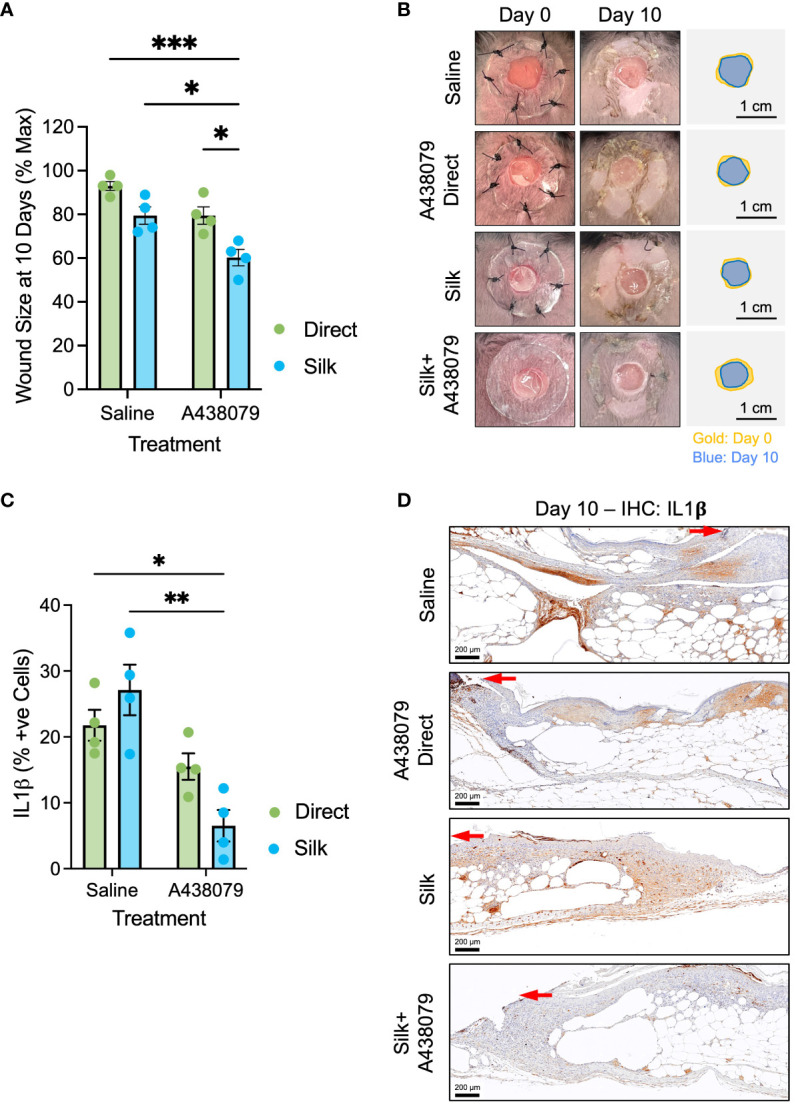
*In vivo* evaluation of A438079-loaded films. **(A)** Planimetry at 10 days post-wounding of db/db mice treated with saline, A438079, silk films, or silk loaded with A438079. Data are given as mean ± standard error and statistics are two-way ANOVA with Sidak multiple comparisons test *p<0.05, ***p<0.001. **(B)** Representative wound images at day 0 and day 10 post-wounding with traces to illustrate degree of closure shown in gold (day 0) and blue (day 10) with 1-cm scale bar. **(C)** Quantification of IL1β positive (+ve) cells at the wound edge on day 10 with the indicated treatments. Data are given as mean ± standard error and statistics are two-way ANOVA with Sidak multiple comparisons test *p<0.01, ***p<0.05. **(D)** Representative immunohistochemistry micrographs of IL1β staining on day 10 post-wound tissues for the indicated treatments. Red arrow terminates at the edge of the migrating epidermis. Scale bars are 200 µm.

We generated full-thickness 6-mm biopsy punch wounds in the dorsum of genetically diabetic db/db mice (**Figure 4**). Wounds were treated at the time of wounding either with saline or saline solution containing 100 µM A438079 without silk film (“direct A438079;” dosage selected according to release dynamics *in vitro* – **Figure 2D**) or had empty silk films or silk films loaded with A438079 applied. Planimetric analysis was performed at 10 days post-wounding, similar to prior reports (19). We found that silk films alone and a topical aqueous solution of A438079 partially, but non-significantly, enhanced wound closure. By comparison, silk films loaded with A438079 resulted in a 40% wound closure which was statistically significant versus saline treated, empty film-treated, and direct drug-treated wounds (**Figures 4A, B**). We performed immunohistochemical staining for IL-1β as the most direct downstream inflammatory mediator of inflammasome activation (**Figures 4C, D**). Quantification of IL-1β positive cells at the wound margin indicated a statistically significant reduction in wounds treated with silk films loaded with A438079 compared to saline or empty silk film treated wounds. Comparison to direct A438079 treated wounds approached significance (p=0.21), suggesting even a day 0 treatment with A438079 exerted some anti-inflammasome activity over the course of diabetic healing. Thus, A438079-loaded films, acting as a depot, promote enhanced and sustained anti-inflammasome activity resulting in improved wound closure in diabetic wounds.

## 4 Discussion

We describe a silk fibroin wound dressing loaded with inflammasome-modulating cargo for use in diabetic wound treatments. Silk fibroin has gained attention as a sustainable, biocompatible, and physicochemically alterable biomaterial amenable to fabrication into sutures, films, and particles for use in wound care (47). By insolubilizing the films with autoclave pretreatment, our films exhibit no loss of integrity by dissolution. However, we cannot exclude the possibility of proteolytic degradation in the wound exudate (48), which may explain the modest and non-significantly enhanced closure by unloaded films (**Figure 4**), as soluble silk fibroin products are known to exhibit immune-stimulating and regenerative properties (49, 50).

We also note a similar non-significant effect of topical direct application of A438079 at the time of wounding, which may have blunted initial responses to ATP released at the time of injury. However, extracellular ATP may be released by post-injury death in cells undergoing apoptosis via pannexin channels or necroptotic rupture (51–53). Thus, the sustained delivery of A438079 in silk films allows prolonged suppression of P2X7 receptor activation and inflammasome activity, as demonstrated by our immunohistochemical finding of reduced IL-1β levels at day 10. This effect may be enhanced further by negative feedback, as inhibition of P2X7-mediated pyroptotic cell death has been shown to suppress subsequent extracellular ATP release (54) and IL-1β signaling positively regulates further expression of IL-1β (55).

A438079 has previously been shown effective in several models of inflammatory disease (56–62). However, despite potent effect, it has been noted that clinical translation may be a challenge owing to its short half-life of 40 minutes (63) to ~1 hour (25). Similarly, prior work on delivering small molecule inflammasome modulators to wounds also required repeated administration (19, 20). Here, we demonstrate that tunable wound dressings which may be stored dry after drug loading are capable of releasing A438079 for several days without repeated administration, providing an appropriate use-case for clinical wound application. By comparison, hydrogel formulations releasing modulators of inflammasome pathway components pose potential challenges for shelf stability and end-user complexity (64). Furthermore, whereas drug-loaded hydrogels require costly reagents, recombinant proteins, and fabrication methods, silk fibroin wound dressings are low cost, sustainable, and can be produced in large-scale batches with minimal difficulty (65).

Several groups have noted that the NLRP3 inflammasome is critical for wound healing. Work by Weinheimer-Haus and colleagues (66), and confirmed by Ito et al. (67), reported that mice deficient in NLRP3 or caspase-1 exhibited reduced epithelialization rates and angiogenesis versus wildtype mice. In this respect, inhibition of inflammasomes may be detrimental to healthy wound healing. By contrast, repeated observation that several comorbidities characterized by delayed healing have been associated with sustained inflammasome activation, including diabetes (19), aging (68), and burns (69, 70), underscores the dichotomous role for this key innate immune pathway in health and disease (71). For example, Tan et al. reported that disruption of IL-1 signaling, using either genetic knockout of the IL-1 receptor or introduction of a recombinant fusion matrix-binding IL-1 receptor antagonist, can stimulate healing in diabetic mouse wounds (64). Thus, for wounds in which inflammasome activity is dysregulated (e.g., diabetic wounds), pharmacologic inhibition may be appropriate. In effect, initial inflammasome signaling facilitates early healing processes, but appropriate control is necessary to negatively regulate the pathway and allow a pro-resolution phenotype to begin. Diabetes and other comorbidities fail to provide this negative regulation, and unchecked sustained activity results in prolonged inflammation and an inability to shift towards a pro-resolution phenotype. The question remains: why is inflammasome activity needed for healthy wound healing, but detrimental in diabetic wound healing? Future work will investigate this dichotomous role for inflammasome signaling with respect to targeted drug delivery.

## Data availability statement

The raw data supporting the conclusions of this article will be made available by the authors, without undue reservation.

## Ethics statement

The animal study was approved by Institutional Animal Care and Use Committee of Arizona State University. The study was conducted in accordance with the local legislation and institutional requirements.

## Author contributions

JY: Conceptualization, Data curation, Formal analysis, Funding acquisition, Investigation, Methodology, Project administration, Resources, Supervision, Visualization, Writing – original draft, Writing – review & editing. SB: Investigation, Methodology, Writing – review & editing. NG: Investigation, Writing – review & editing. FB: Investigation, Writing – review & editing. SM: Investigation, Writing – review & editing. SR: Investigation, Writing – review & editing. SN-D: Investigation, Writing – review & editing. KR: Conceptualization, Funding acquisition, Project administration, Resources, Supervision, Writing – review & editing.
