# Supplementary material for: Inflammasome modulation with P2X7 inhibitor A438079-loaded dressings for diabetic wound healing
Source: Front Immunol. 2024 Feb 15;15:1340405. doi: 10.3389/fimmu.2024.1340405 (PMC10901979; doi:10.3389/fimmu.2024.1340405)
Supplement: Supplementary file 1 [file DataSheet_1.docx]

**Supplemental Information**

**Inflammasome Modulation with P2X7 Inhibitor A438079-loaded Dressings for Diabetic Wound Healing**

Jordan R. Yaron^a,b*^, Selin Bakkaloglu^a^, Nicole A. Grigaitis^a,c^, Farhan H. Babur^a^, Sophia Macko^a^, Samantha Rhodes^a^, Solenne Norvor-Davis^a^, Kaushal Rege^a,b,c,d^

*^a^ Center for Biomaterials Innovation and Translation, The Biodesign Institute, Arizona State University, Tempe, AZ*

*^b^ School for Engineering of Matter, Transport & Energy, Arizona State University, Tempe, AZ*

*^c^ Biological Design Graduate Program, Arizona State University, Tempe, AZ*

*^d^ Chemical Engineering, Arizona State University, Tempe, AZ*

Correspondence:

Jordan R. Yaron, PhD

Center for Biomaterials Innovation and Translation

650 E. Tyler St. GWC 682

Tempe, Arizona 85287 USA

Email: [jyaron@asu.edu](mailto:jyaron@asu.edu)

Phone: 480-231-4334

**Supplemental Figures**

**Figure S1. Evaluation of loading time effects on A438079 release dynamics from 60-µm thick silk films.** Data are given as mean ± standard error. Statistics are calculated by unpaired T-test per day. *****p*<0.0001 indicating day-to-day statistical significance of enhanced drug release with 3 days of loading versus 1 day.

**Figure S2. Non-fasting blood glucose levels of db/db mice prior to surgery.** Non-fasting blood glucose measured in male (N=4) and female (N=4) mice 1 week prior to wound surgery. Mean ± SEM shown along with data points. Statistical analysis performed by unpaired T-test. Diabetic threshold level of 300 mg/dL represented by dotted red line.
